# Assessing renal function in children with hydronephrosis – additional feature of MR urography

**DOI:** 10.2478/v10019-011-0038-z

**Published:** 2011-11-16

**Authors:** George Hadjidekov, Savina Hadjidekova, Zahari Tonchev, Rumiana Bakalova, Ichio Aoki

**Affiliations:** 1 Department of Radiology, University Hospital “Lozenets”, Sofia, Bulgaria; 2 Department of Physics, Biophysics & Radiology, Medical Faculty, University Sofia University “St. Kl. Ohridski”, Sofia, Bulgaria; 3 Department of Medical Genetics, Medical University, Sofia, Bulgaria; 4 Molecular Imaging Center, National Institute of Radiological Sciences (NIRS), Chiba, Japan

**Keywords:** MR urography, children, functional analysis, urinary tract

## Abstract

**Background:**

Magnetic resonance urography (MRU) is one of the most attractive imaging modalities in paediatric urology, providing largest diagnostic information in a single protocol. Therefore, the aim of our study was to assess the diagnostic value of MRU in children with urogenital anomalies (especially anomalies of the renal pelvis and ureter) and the renal function using different post-processing functional software.

**Patients and methods:**

Ninety six children (7 days – 18 years old) were examined. In 54 patients of them, a static T_2_ MRU was completed by excretory T_1_ MRU after gadolinium administration and functional analysis has been performed using two functional analysis softwares “CHOP-fMRU” and “ImageJ” software.

**Results:**

MRU showed suspicious renal and the whole urinary tract anomalies with excellent image quality in all children. In ureteropelvic obstruction, MRU was confirmatory to the other imaging techniques, but it was superior modality concerning the evaluation of end-ureteral anomalies. There was an excellent correlation between the MRU data and diagnosis, determined by surgery. The renal transit times, renal volumes and volumetric differential renal function were assessed separately by “CHOP-fMRU” and “ImageJ” with excellent agreement with 99^m^Tc-DTPA and among them.

**Conclusions:**

MRU overcomes a lot of limitations of conventional imaging modalities and has a potential to become a leading modality in paediatric uroradiology. Synthesis of both anatomical and functional criteria in MR urography enables to select the best candidates for surgical treatment. Even small kidney dysfunction can be detected by functional analysis software.

## Introduction

The imaging of urinary tract is important clue in paediatrics. Different methods for evaluation of the genitourinary system are routinely used in the clinic. However, there is no single method providing the whole information, necessary for the diagnostic. The conventional methods have many limitations. For example: ultrasound examination is operator-dependant, with sometimes difficult visualization of the end-ureter; in intravenous urography, there is a risk of contrast media and ionizing radiation; retrograde methods are invasive with limited application; scintigraphy has a poor anatomical resolution.^1^

Novel methods have developed to overcome the limitations of the conventional methods and MR urography (MRU) is one of the most attractive. MRU is a promising method for early diagnosis, having an impact on the management of congenital malformations and other urogenital anomalies in children.^1^ This diagnostic modality provides a detailed visualization of various morphologic abnormalities of the genitourinary system and avoids radiation, which is mutagenic.^1^,^2^ To avoid ionizing radiation is one of the most important diagnostic approaches in children.^3^

Currently, MRI is used in paediatrics for assessment of the congenital abnormalities of the genitourinary system, different cases of obstruction of the excretory system and evaluation of renal tumours, which are prevalent solid tumours in infants.^1^,^4^ In addition to the morphological imaging, MRI can be used to quantify the renal function. Following contrast administration and using appropriate software, time-intensity curves can be generated and other parameters (*e.g*., renal transit times, renal volumes and differential renal function) can be quantified.^1^ This is the reason some authors to define MRI as a potential “one-stop-shop” imaging technique for a variety of renal diseases.^6^–^8^

In the present study, we assess the diagnostic value of MRU in a cohort of paediatric patients with various urogenital anomalies (especially with anomalies of the renal pelvis and ureter) using two post-processing functional software “CHOP-fMRU” and ImageJ and in comparison to 99^m^Tc-DTPA scintigraphy.

## Materials and methods

### Patient population

We retrospectively reviewed all 96 children (age: between 7 days and 18 years) referred from the Department of Urology and Paediatrics, between 2006 and 2010 with suspected congenital urinary tract anomalies, controversial findings from the conventional imaging studies and difficulties to establish the final diagnosis. In 54 of them an excretory, T_1_ MRU after contrast administration of gadolinium has been performed for renal function assessment in addition to T2 MR urography. In the remaining 42 patients, static T2 MR urography has been employed in order to confirm conditions affecting the urinary tract without impact on the renal function, co-existing renal pathology or due to contraindications for gadolinium (Gd) injection in cases of renal failure. The frequency of age distribution in the patient population was as follows: 0 day – 1 month: 7 patients (7.3% from the whole study group of 96 patients); 1 month – 1 year: 29 patients (30.2%); 1 year – 6 years: 18 patients (18.8%); 6 years – 14 years: 15 patients (15.6%); 14 years – 18 years: 27 patients (28.1%).

Cross-sectional sequences, MR angiography in the arterial and venous phase, serial evaluation of the renal parenchymal perfusion and contrast-enhanced MRU were combined in one imaging session instead of lining up several different imaging modalities. Time-intensity curves were generated, based on the dynamic 3D post-contrast sequences. “CHOP-fMRU” and ImageJ analysis software was used for calculation the functional curves, plots and maps, renal transit times, renal volumes and differential renal function.^5^,^10^ In all cases, an informed consent was obtained after the procedure was fully explained to the parents and older children and the study was approved by the Ethics Committee of the University Hospital “Lozenets”, Sofia, Bulgaria.

Ultrasonography was conducted in all patients prior to MRU examination. Voiding cystourethrography (VCUG) was performed in 10 children with suspicion for dilatation of urinary tract in accordance to vesicoureteral reflux (VUR). In 8 children intravenous urography (IVU) has been previously done and in 19 cases 99^m^Tc-DTPA scintigraphy as a part of the uroradiological work-up has been done with a delay prior or after the MRI exam no longer than 1 month, in another institution. The 99^m^Tc-DTPA protocol was similar to our MRU protocol in terms of hydration with intravenous administration of 10 ml/kg sodium chloride solution 30 min prior to the scan. The amount furosemide (1 mg/kg, *i.v.*) was the same as in our examination, although diuretics have been given when maximum pelvicalyceal distension was observed (usually 10–15 min after administration of 99mTc-DTPA).

### Patient preparation

The adequate preparation is a prerequisite for a good image quality.^5^–^11^ We didn’t place routinely a bladder catheter, although catheterisation of small children is recommended in case of megaureter (with or without reflux). We used catheter in few patients with suspected VUR, but due to technical problems we abandoned this procedure. Then we started to scan without catheterization and we were happy with cooperative, toilet-trained children, without cases of severe discomfort or inability to conduct the examination. The intravenous hydration and administration of furosemide are crucial for reducing the concentrations of Gd.^10^ Diuretics are recommended in both static urography and dynamic urography before contrast administration. In this context, we administered standardised hydration (10–15 ml/kg sodium chloride or Ringer’s solution) and diuretics (furosemide – 1 mg/kg, max. dose 20 mg) 15 min prior to Gd injection, in order to reduce artefacts, to distend the urinary tract, to maintain the linearity between signal intensity and concentration of Gd and to shorten the examination time, adopting the F-15 protocol, proposed by Grattan-Smith.^12^ In children younger than 6-year-old and non-cooperative for breath-hold techniques, successful sedation was performed with ketamine (Ketalar) and midazolam (Dormicum) according to the department’s standard sedation protocol with no serious adverse effects. In 15 patients intravenous sedation (Ketalar – 1 mg/kg or Thiopental 4–5 mg/kg) was successfully performed with minor motion artefacts in 2 infants without any impact on the diagnostic value of the image quality. Oral sedation using midazolam (Dormicum - 0.5 mg/kg) was sufficient to perform MRU with excellent diagnostic image quality in 32 patients and there was no major complaint of nausea and vomiting that could be related to antiemetic effects of midazolam.^13^–^14^ The blood pressure, respiration, heart rate, and oxygen saturation were continuously monitored throughout MR imaging in all patients.

### MRU protocol

**FIGURE 1A–C:**
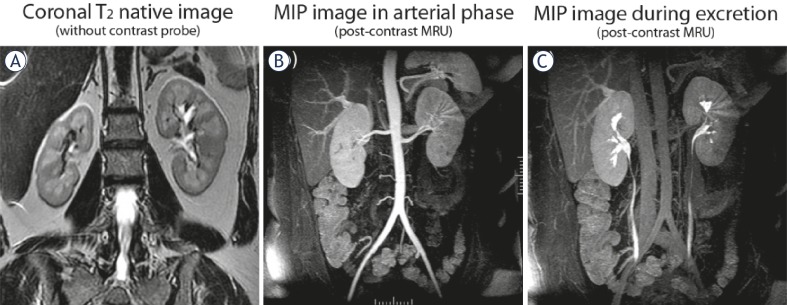
Normal MR urogram in 5-year-old boy. **A.** Coronal T2 native image of both kidneys. **B,C.** MIP images from two separated time-points of the excretory post-contrast MRU in arterial phase (B) and during excretion (C).

High-field strength tomographs (1.5 Tesla) (Signa, General Electric Medical Systems and Magnetom Essenza, Siemens Medical Solutions) were used with large field of view (FOV) above diaphragm to avoid artefacts from aliasing or post-contrast signal intensity decline in the upper renal poles and obtain an oblique coronal plan angled parallel to the long axis of the kidneys, including ureters and bladder. Our MRU protocol consisted of native MR examination with T_2_ coronal, T_1_ and T_2_ axial sequences, followed by dynamic study with Gd injection, administration of furosemide prior to the dynamic acquisitions and 3D reconstructions. Following the coronal T_2_ plan, we performed axial T_2_ and T_1_ sequences. Fat-suppression techniques were used in T_1_ and T_2_ hyperintense findings and in cases of suspicion of tumour formation – In/Out phase dual-echo sequences for contour delineation. The most important pre-contrast sequence was 3D T_2_ urogram with fat-suppression and respiratory-triggering. T_1_-weighted gradient-echo sequence with fat-saturation (3D Dyn SPGR for GE 1.5 T Signa and 3D VIBE Dynamic for Siemens Essenza 1,5T) was used for the post-contrast scan. The dynamic scan was repeated within 15 min, following Gd injection with increasing intervals between acquisitions, for the need of post-processing. Our sequences were compatible on both MR units and the software used for post-processing has been properly validated for correctness and applicability in our MR protocols. We employed a standard dose of 0.1 mmol/kg of Gd in the majority of our studies, however in some occasions low-dose Gd opacification – 0.01 mmol/kg has been employed, especially in small infants and in cases of glomerular filtration between 30 and 60 ml/min/1.73 m^2^. In all our patients, serum creatinine levels were strictly observed and we estimated individually the glomerular filtration rate according to the Schwartz’s formula.^15^ New-borns and small infants were scanned with a head-coil and the older children were scanned with a phased-array torso coil. Normal MR urogram is shown in Figure 1.

### Statistical analysis and ethical consideration

Groups were compared with Mann-Whitney *U*-test, *P*-values >0.05 were taken as indicators of no statistically significant differences. SPSS 13.0 (SPSS Inc., Chicago, Illinois, USA) was used.

The investigators strictly followed recommendations of the Helsinki Declaration (1964, with later amendments) and of the European Council Convention on Protection of Human Rights in Bio-Medicine (Oviedo 1997).

## Results

### MR urography for visualization of morphological renal anomalies in children

**FIGURE 2A–I:**
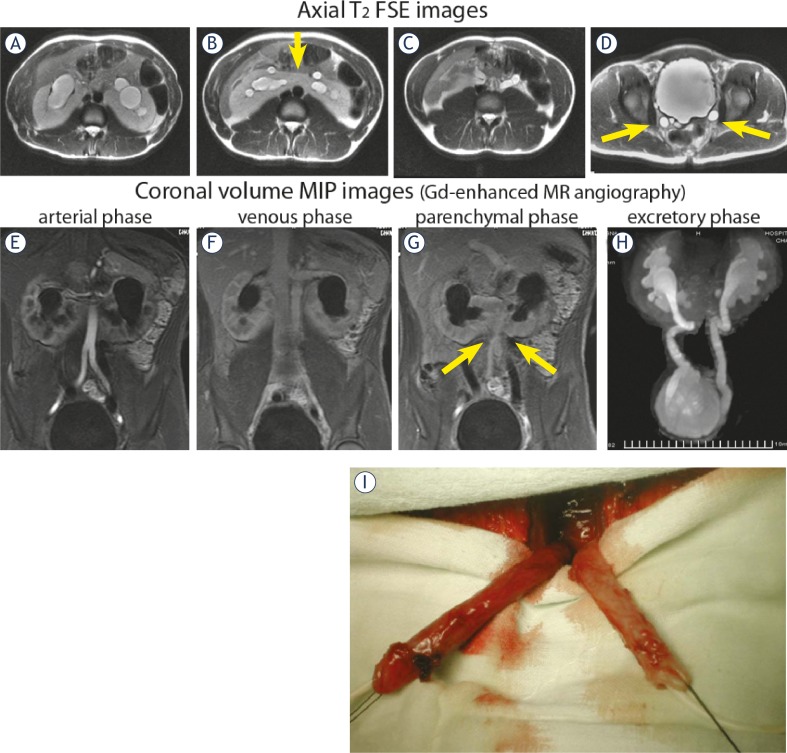
MR imaging of horseshoe kidney in 12-year-old boy. **A-D.** Axial T2 FSE images – clearly dilated pyelocalyceal system and ureters in horseshoe kidney. **E.** Coronal volume MIP image from arterial phase of 3D Gd-enhanced MR angiography – the main left and right renal arteries extending from the anterior aspect of the aorta. **F.** Coronal volume MIP image from venous phase – both renal veins in their expected locations. **G.** Coronal volume MIP image from parenchymal phase – lower poles of the kidneys without any parenchymal abnormalities. **H.** Coronal volume MIP image from excretory phase – marked dilatation of both pyelocalyceal systems and ureters. **I.** Intraoperative findings prove the diagnosis of bilateral megaureters in horseshoe kidney with dysplastic changes in their distal thirds.

Static, T2 MR urography was successfully performed in 96 children with 99 exams, totally 197 kidneys (in three children follow-up MRI exams after surgery have been done and in one patient left nephrectomy was performed). T2 images for anatomic evaluation were helpful in the assessment of children with severe hydronephrosis and poorly functioning systems. The majority of the population (91 cases) presented with congenital anomalies of the renal pelvis and ureter, including megacalycosis, ureteropelvic (UPJ) obstruction and primary megaureters. We also detected 36 cases of congenital anomalies of the kidney, including: renal agenesis – 6; renal hypoplasia – 5; cystic anomalies of the kidneys – 8; anomalies of renal rotation, horseshoe kidney – 6 (Figure 2); renal dystopia – 3; abnormal renal vessels – 6; Fraley’s syndrome -2. Static, T2 MRU allows us also to find the following anomalies: (i) bladder anomalies – in 3 children; (ii) encountered lower urinary tract anomalies of urogenital sinus – in 7 children, including disorders of sex development with ambiguous genitalia (hermaphroditism) (n=3), anorectal and vaginal malformations (n=4); (iii) renal infections – in 18 children. 11 cases of renal neoplasms were confirmed or detected on MRU. In 13 cases, no abnormalities were detected on the static, T2 MR urography.

### MR urography for assessment of renal function in children

**FIGURE 3A–C:**
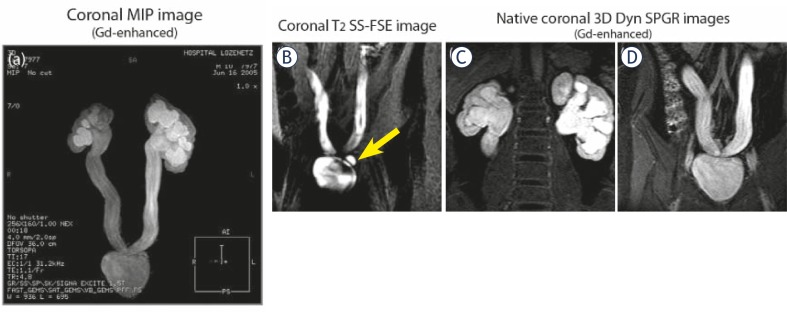
MR imaging of bilateral ureterocystoneostomies in 10-months old boy. **A.** Coronal post-contrast MIP image – persistent bilateral hydronephrosis and hydroureters following surgery. **B.** Coronal T2 SS-FSE image – a bladder diverticula. **C,D.** Post-contrast coronal 3D Dyn SPGR image on the kidney level (C) and on the level of the bladder (D) – re-implanted dilated hydroureters.

In 54 children (from the whole population), T1 excretory MR urography with injection of Gd has been performed in addition to static, T2 MR urography for the main purpose of our study – to assess the renal function. The majority of them had anomalies of the renal pelvis and ureter: ureteropelvic (UPJ) obstruction (hydronephrosis) – 43 (bilateral – 10, right side – 14, left side – 19); primary megaureter and anomalies of vesicoureteral segment (UVJ) – 30 (bilateral – 8, right side – 8, left side – 14) including 7 patients with vesicoureteric reflux (VUR), diagnosed by VCUG, ureter duplication – 2; ureterocele – 2. We observed obstructed systems on MR urography morphologically by the presence of narrowed ureter with proximal dilatation and we were able to distinguish obstructed from non-obstructed systems functionally by the presence of delayed contrast excretion into the collecting system and ureter on the basis of the functional analysis in particular by the calculation of renal transit times (RTT). In 40 children MR functional analysis proved the presence of obstructive systems and the remaining 14 children were classified as non-obstructive and they have been followed-up. Both static and excretory MR urography was helpful in differentiating the causes of hydronephrosis in these patients. Typical images of a child with several bilateral ureterocystoneostomies and persistent bilateral hydronephrosis and hydroureters following surgery are shown in Figure 3.

We consider images quality of the kidney and the collecting system to be superior with MR urography in comparison to ultrasound and DTPA renogram in all 96 cases. The agreement of grading of hydronephrosis was equal in MR urography and ultrasound (US), however MR provides a detailed visualization of the entire ureters and presents ureteric pathology clearly US.

**FIGURE 4A–G:**
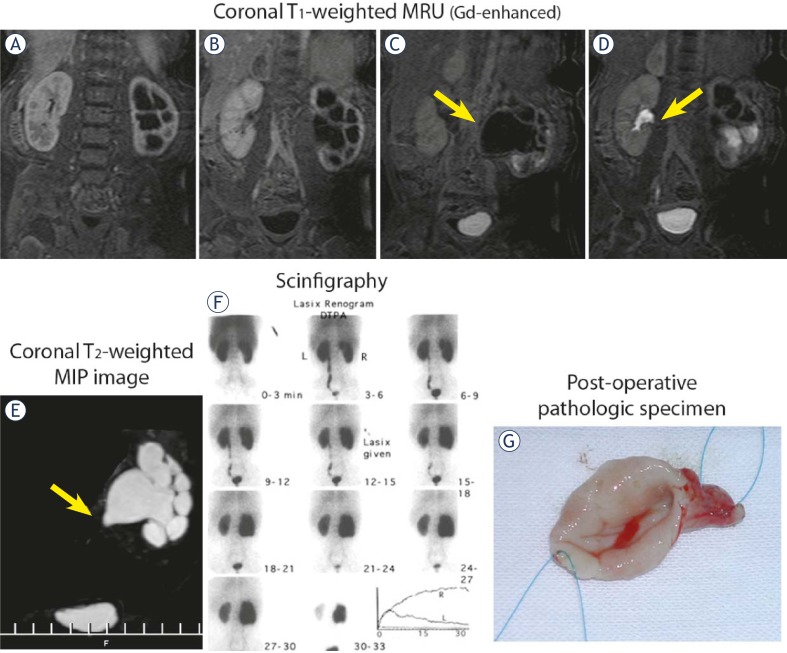
Imaging of UPJ obstruction in 9-month-old boy. **A–D.** Consecutive coronal T1-weighted MR images (Gd-enhanced) – successively filling of the right renal pelvis with preservation of the right kidney function. **E.** Coronal T2-weighted MIP image – on the left side an outflow tract obstruction with marked dilatation of the left pyelocalyceal system; **F.** Dynamic 99mTc-DTPA presenting urinary obstruction of the left kidney; **G.** Postoperative pathologic specimen in th same child following pyeloplasty a modo Anderson-Hynes.

A correlation between MRU data and final diagnosis determined by surgery or observation was excellent in all 96 patients. 40 children benefits from surgical interventions for obstructive systems. Pyeloplasty has been performed in 11 with MR findings of ureteropelvic junction (UPJ) obstruction (Figure 4). In 29 children with UVJ obstruction and primary megaureter, reimplantation of the ureters - ureterocystoneostomy (UCNS) has been performed (Figure 3). Other surgical interventions (74 in total for the whole study population), such as nephrectomies, partial or atypical kidney resection, nephrostomies, external genitalia corrections, masculinizing surgical procedures, retroperitoneal tumours resections etc. were also confirmed at MR urography.

VCUG was performed in 10 patients. Vesico-ureteric reflux (VUR) in 7 patients and in one case an ureterocele was identified. The vesico-ureteric reflux was classified as grade III in 4 children, grade IV in 2 and grade V in 1; in both cases presenting dilatation of the ureter and the pyelocalyceal system were clearly visible on MR urograms. In two cases VCUG present normal findings.

**FIGURE 5A–C:**
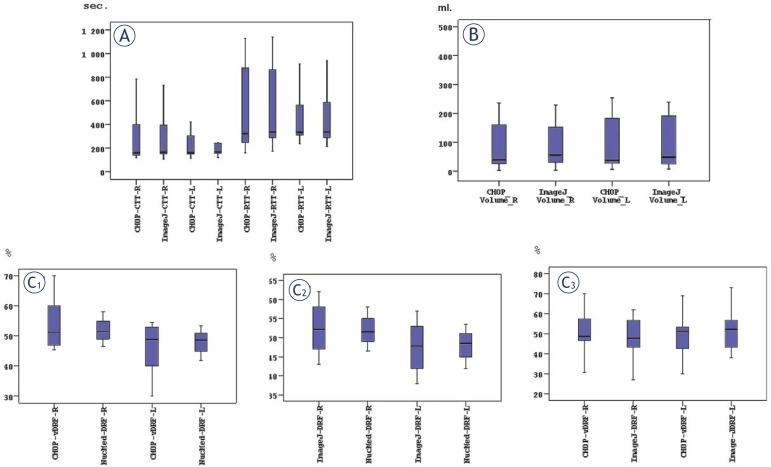
Box-plots of different parameters for right and left kidney evaluated by CHOP-fMRU and ImageJ. **A.** cTT and rTT. **B.** Parenchymal volumes. **C1,2,3.** Volumetric differential renal function, as well as 99mTc-DTPA renal function.

A comparison of the results from the functional analysis has been done by two different softwares – “CHOP-fMRU” and “ImageJ”, as well as by the data from the 99mTc-DTPA. The results from the functional analysis of transit times, volumes and volumetric differential renal function are presented on Table 1. No statistically significant differences (*P*>0.05) were found between the calyceal and renal transit times and the parenchymal kidney volumes, measured by CHOP-fMRU and ImageJ (Figure 5A,B). The values for the volumetric differential kidney function assessed by CHOP-fMRU and ImageJ measured separately for each kidney were not statistically different to those derived from the Tc-DTPA study (*P*>0.05) (Figure 5C**_1,2,3_**). MR urography and renal scintigraphy showed confirmatory results in the diagnosis of obstruction both UPJ and UVJ in terms of volumetric differential renal function values.

## Discussion

MRU is a feasible method for evaluation of urinary tract pathology in neonates and infants.^1^,^16^ It overcomes the limitations of the conventional imaging techniques and is superior tool in many aspects, especially in the evaluation of parenchymal kidney diseases and poorly functioning systems, assessment of ureteral anatomy and renal vasculature as shown in our study. The method is particularly helpful for further therapeutic decisions, planning of surgical intervention and future diagnostic work-up.

**FIGURE 6A–C:**
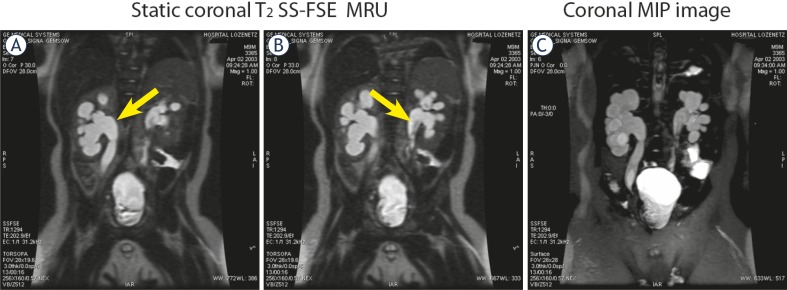
MR imaging of persistent bilateral hydronephrosis and hydroureters in 9-month old boy, following ureterocystoneostomy. **A,B.** Static coronal T2-weghted MR images using single-shot fast spin echo (SS-FSE). **C.** Coronal MIP image.

The most common MRU techniques, used to visualize the urinary tract, are the static (T_2_) MRU and excretory (T_1_) MRU.^17^–^19^ Three-dimensional (3D) sequences are used to obtain thin-section data sets that can be further post-processed to create volume-rendered (VR) or maximum-intensity-projection (MIP) images of the entire urinary tract (Figure 6). Similar observations have been as reported by Roy *et al.* and O’Malley *et al*., using MRU.^20^–^21^ Excretory (T_1_) MRU is similar to CT urography and intravenous urography. The use of dose of Gd (0.1 mmol/kg) and in some occasions low-dose Gd opacification (0.01 mmol/kg) allowed us to maintain the linearity between signal and Gd concentration, which is essential for quantitative measurements and analysis. Administration of diuretics improved the quality of MRU by increasing the quantity of the urine and therefore, leads to better dilution and appropriate distribution of Gd in the urinary tract.^22^–^23^ The most important sequence of excretory MRU in our study was 3D gradient-echo. Fat-suppression is recommended for better demonstration of the ureters. Modern MR-units scan simultaneously in one volume the kidneys, the ureters and the bladder, using 3D gradient-echo sequences in one breath-hold.^19^,^24^ Sometimes segmental scanning of the kidneys or bladder separately for visualization of image details is recommended.

Currently, there are two major MRU processing software available free of charge, which we have verified, compared each other and used in our practice routinely.^5^,^9^ Post-processing algorithms permits us to evaluate and compare to scintigraphy several parameters – (i) calycial (cTT) and renal transit times (rTT); (ii) parenchymal volumes; (iii) differentiated renal function (vDRF) and (iv) the time-intensity curves representative for the renal function.

Our results demonstrate that MRU should be a method of choice for visualization of the upper urinary tract in children as it is difficult to assess by US or scintigraphy. In some cases, such as UPJ obstruction, MRU was confirmatory to ultrasound, but superior concerning the evaluation of endureteral anomalies. US provides initial information concerning renal parenchyma, bladder morphology, presence and degree of dilated systems, but failed in visualization of non-accessible ureters, hidden in the retroperitoneum and is pretty week in information about renal excretion.

The graphic presentation of time-intensity curves, obtained by the dynamic MRU studies, was in accordance with the renal curves, obtained by scintigraphy. Moreover, the calculated values of the volumetric differential renal function, using “CHOP-fMRU” software were similar to those calculated on the basis of ImageJ software; both corresponded to the values from the dynamic 99^m^Tc-DTPA scintigraphy. Comparable results regarding parenchymal volumes and transit times were observed using the two different software programs. Scintigraphy also supplies information about the renal function and morphology; however it is time-consuming ionizing imaging method with low spatial resolution.^6^,^25^ In our study, the basic parameters of the curves (amplitude, washout) were assessed, as well as the presence of certain characteristic features of the curve. The data from the “signal-intensity versus time-curve” analysis were combined with the other parameters, derived from the MRU analysis, including estimation of the renal transit times, parenchymal volumes and differential renal function. The resulted data-set provided a powerful tool, of high importance for the diagnosis of obstruction.

**FIGURE 7A–E:**
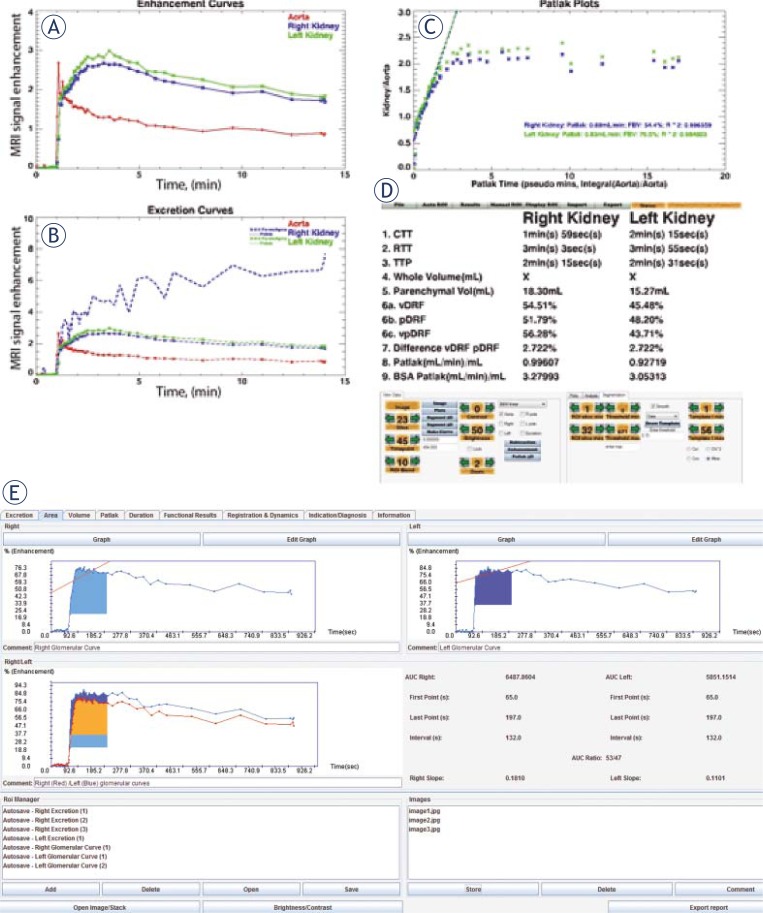
Automated functional analysis of MRU data in bilateral normal kidney with vesicoureteral reflux(VUR) – grade 1 on the right side using “CHOP-fMRU”. **A.** Enhancement curves. **B.** Excretion curves. **C.** Patlak plots. **D.** Calculation of renal transit times and differential renal function. **E.** Enhancement curves, generated on “ImageJ”.

In the data processing, several parameters were also calculated, using “CHOP-fMRU” and “ImageJ”: CTT – calyceal transit time; RTsT – renal transit time; TTP – time to peak; parenchymal volume; vDRF – volumetric differential renal function; pDRF – Patlak differential renal function etc. Typical example of data processing is shown in Figure 7 – a child with recurrent renal infections and a low-grade vesicoureteral reflux (VUR) on the left side. On non-contrast MRU images dilatation of the distal part of the left ureter was observed, the resulting enhancement curves were non-obstructive and the patient was referred to ultrasonography follow-up. No significant difference concerning the listed parameters was found whatever functional analysis software has been used.

Our results as well as the presented case (Figure 7) showed that both renal and calycial transit times, parenchymal volumes and differential renal function are indicators for kidney dysfunction. Even small functional disorders can be detected using MRU and analysing these parameters. The complex software functional analysis of the whole patient population confirmed this assumption.

In addition to the advantages of MRU, mentioned above, it is necessary to note that this technique has also some limitations. Sometimes it requires a placement of bladder catheter, administration of furosemide and Gd, sedation and even anaesthesia (for newborns and younger children), as a complementary risk. Breath-hold techniques could not be applied in neonates and small infants and motion artefacts should be at a minimum. Patient preparation and examination itself are time-consuming; post-processing and calculation of functional curves and differential renal function requires additional time.

In 2006, it was demonstrated that some Gd-based contrast agents may provoke the development of nephrogenic systemic fibrosis (NSF) and/or a generalized fibrotic disorder in renal failure patients.^26^ Gd-ions, released from Gd-based MR contrast agents, are the likely etiologic agent of NSF.^27^ The ESUR guidelines suggest a very careful administration of Gd in children with renal failure. Absolute contraindications are high levels of creatinine and a glomerular filtration under 30 ml/min/1.73 m^2^. Individual assessment for the indications and the need of contrast-enhanced MR examination was performed after discussions with paediatric nephrologists in cases of glomerular filtration between 30 and 60 ml/min/1.73 m^2^. Written consent should be obtained in spite of the fact that most cases of NSF occurs in adults and the reported cases of NSF without Gd administration. In all patients with high risk for development of NSF and in the paediatric group, we used cyclic Gd-helators due to their higher stability.^28^ We didn’t observe any adverse effects or cases of NSF, following contrast administration in our study-group.

In conclusion, MR urography is useful, non-ionizing method for assessment of obstructive uropathies and facilitates surgical decisions. There is growing number of publications concerning the criteria for assessment of the renal function in children by dynamic MRU, but the achievement of consensus requires more and deeper investigations. The advances of molecular imaging techniques provide new insights about the nature of hereditary diseases in paediatric nephrology and urology.

## Figures and Tables

**TABLE 1:** Calculated transit times, parenchymal volumes and volumetric differential renal function

**Transit times**		**Parenchymal volumes**		**Volumetric differential renal function**			

**CHOP-fMRU/ImageJ**	**Time (range)**	**CHOP-fMRU/ImageJ**	**Volume (range)**	**CHOP-fMRU/ImageJ/NucMed**	**Percent (range)**	**SE**	**SD**
CHOP-CTT-R	313 sec. (150–476)	CHOP-Volume-R	134,9 ml (14,3–255,6)	CHOP-vDRF-R	54,15% (44,18–64,11)	3,88	9,50
ImageJ-CTT-R	279 sec. (151–407)	ImageJ-Volume-R	129,2 ml (19,5–238,9)	CHOP-vDRF-L	48,85% (35,88–55,82)	3,88	9,50
CHOP-CTT-L	267 sec. (141–393)	CHOP-Volume-L	147,2 ml (12,4–282,0)	ImageJ-DRF-R	52,40% (44,60–60,20)	3,04	7,44
ImageJ-CTT-L	243 sec. (126–361)	ImageJ-Volume-L	150,3 ml (15,7–284,9)	ImageJ-DRF-L	47,60% (39,80–55,40)	3,04	7,44
CHOP-RTT-R	534 sec. (287–780)			NucMed-DRF-R	51,92% (47,27–56,56)	1,81	4,43
ImageJ-RTT-R	550 sec. (306–793)			NucMed-DRF-L	48,08% (43,44–52,73)	1,81	4,43
CHOP-RTT-L	476 sec. (290–663)						
ImageJ-RTT-L	475 sec. (277–673)5						

Legend: R = right kidney, L = left kidney; CHOP-CTT = mean calycial transit time measured with CHOP-fMRU; CHOP-RTT = mean renal transit time measured with CHOP-fMRU;, ImageJ-CTT - mean calycial transit time measured with ImageJ; ImageJ-RTT = mean renal transit time measured with ImageJ; CHOP-Volume and ImageJ-Volume = parenchymal volumes, measured with CHOP-fMRU and ImageJ; CHOP-vDRF, ImageJ-vDRF and NucMed-DRF = volumetric differential renal function, measured respir. with CHOP-fMRU, ImageJ and Nuclear Medicine; SE = standard error; SD = standard deviation.
